# Gait Changes Vary among Horses with Naturally Occurring Osteoarthritis Following Intra-articular Administration of Autologous Platelet-Rich Plasma

**DOI:** 10.3389/fvets.2016.00029

**Published:** 2016-04-13

**Authors:** Mustajab H. Mirza, Prakash Bommala, Heather A. Richbourg, Nathalie Rademacher, Michael T. Kearney, Mandi J. Lopez

**Affiliations:** ^1^Department of Veterinary Clinical Sciences, School of Veterinary Medicine, Louisiana State University, Baton Rouge, LA, USA; ^2^Department of Pathobiological Sciences, School of Veterinary Medicine, Louisiana State University, Baton Rouge, LA, USA

**Keywords:** kinetics, joint, platelet, equine, lameness, animal, cell therapy

## Abstract

Mechanisms to reduce lameness associated with osteoarthritis (OA) are vital to equine health and performance. This study was designed to quantify response to autologous, intra-articular platelet-rich plasma (PRP) in horses with OA. Kinetic gait analysis was performed on 12 horses with unilateral forelimb lameness and OA in the same limb before and after intra-articular anesthesia (IAA). Radiographs and kinetic data were obtained before and 6 and 16 weeks after PRP administration to same joint, 4 weeks after IAA. Statistical evaluations included filtration effect on platelet concentration, relationship between kinetic variable changes after IAA versus PRP in the affected limb, and associations between response to PRP and response to IAA, platelet concentration, and radiographic OA. A positive response to IAA or PRP was defined as ≥5% improvement in peak vertical force, vertical impulse, or breaking impulse of the affected limb. Out of 10 horses that responded to IAA, 3 responded to PRP at both time points and 4 responded at one. Of the two horses that did not respond to IAA, one responded to PRP at both time points. Filtration increased platelet concentration significantly. The relationship between kinetic variable alterations of the affected limb after IAA and PRP was not significant, and response to PRP was not associated with response to IAA, platelet concentration, or radiographic OA. Changes in kinetic variables following IAA in joints with naturally occurring OA provide a custom standard to assess intra-articular therapy. Kinetic gait changes after intra-articular PRP are variable in horses with moderate to severe forelimb OA.

## Introduction

Joint pain from osteoarthritis (OA) accounts for over 60% of equine lameness (1). The economic impact of lameness is substantial; annual direct and indirect costs are as high as $1 billion per year in the United States horse population of over 9.2 million (2). There is no known cure or gold standard treatment for OA. Signs are often managed with various combinations of systemic and local therapies that include non-steroidal anti-inflammatory drugs, chondroprotectants, corticosteroids, homeopathic supplements, and blood derivatives (1). Oral and injectable treatments have inconsistent results, potential side effects, and do not stop disease progression (3, 4).

Over the last few decades, platelet-rich plasma (PRP) has become increasingly popular to treat musculoskeletal damage and degeneration (5). Platelet growth factors are reported to enhance *in vivo* articular cartilage regeneration, but clinical efficacy is inconsistent (6). Variable response to PRP therapy has been attributed to distinct isolation and preparation methods that impact platelet concentration and quality, as well as filtrate composition (5, 6). Additionally, inconsistent treatment response may stem from differences among stages of naturally occurring OA that are distinct from each other and artificial models of joint trauma (7).

The overarching objective of this study was to evaluate the effect of autologous PRP on lameness in a population of horses with naturally, occurring forelimb OA. An initial goal was to establish a repeatable mechanism to objectively assess gait changes in the population by identifying kinetic gait variables that improved by a minimum of 5% from baseline within the majority of study subjects that exhibited reduced lameness following intra-articular anesthesia (IAA). Hence, the first hypothesis was that kinetic variables change in a consistent pattern among horses that exhibit reduced lameness following IAA in forelimb joints with naturally occurring OA (Table 1) (8, 9). The findings were applied to test the second hypothesis that kinetic gait variables change in the same magnitude and direction following IAA and autologous PRP administration in horses with naturally occurring, forelimb OA.

**Table 1:** **Ground reaction force variables assessed for changes following intra-articular anesthesia and platelet-rich plasma therapy**.

Abbreviation	Variable
PVF_Z_	Peak vertical force
IMP_Z_	Vertical impulse
PFB	Peak breaking force
PFP	Peak propulsion force
IMP_B_	Braking impulse
IMP_P_	Propulsion impulse
ML	Mean loading rate
MU	Mean unloading rate
L_max_	Maximum loading rate
U_max_	Maximum unloading rate

## Materials and Methods

### Inclusion Criteria

A protocol (#10-092) was approved by the Louisiana State University (LSU) Institutional Animal Care and Use Committee prior to study initiation. Horses from the LSU Equine Health Studies Program research herd were evaluated for inclusion in the study using the following criteria: (1) adult horses of either sex, (2) a subjective American Association of Equine Practitioners (AAEP) lameness grade of 2 or 3 in a single forelimb, and (3) confirmed moderate to severe radiographic OA in the fetlock or carpus of the affected forelimb. All horses received a complete physical exam, and lameness was subjectively scored by a licensed veterinarian according to the AAEP lameness scale (10). Radiographic changes in the carpus or fetlock of the affected limb were scored according to published parameters (metacarpophalangeal joint) (11) or a custom scoring system (carpometacarpal joint; Table 2) by a board certified veterinary radiologist. Horses were sedated with 5 mg/kg xylazine IV (Lloyd, Inc., Shenandoah, IA, USA) for standard, orthogonal radiographs prior to inclusion as well as 6 and 16 weeks after PRP treatment.

**Table 2:** **Radiographic carpal osteoarthritis (OA) severity scoring system**.

Radiographic changes	OA score
Intermediate or radial carpal bone corner or chip fragments	3
Osteoarthritis signs	3
Third carpal bone sclerosis	3
Corner or chip fragment and osteoarthritis signs	4
Slab fracture third carpal bone	5

### Study Cohorts

**Figure 1:**
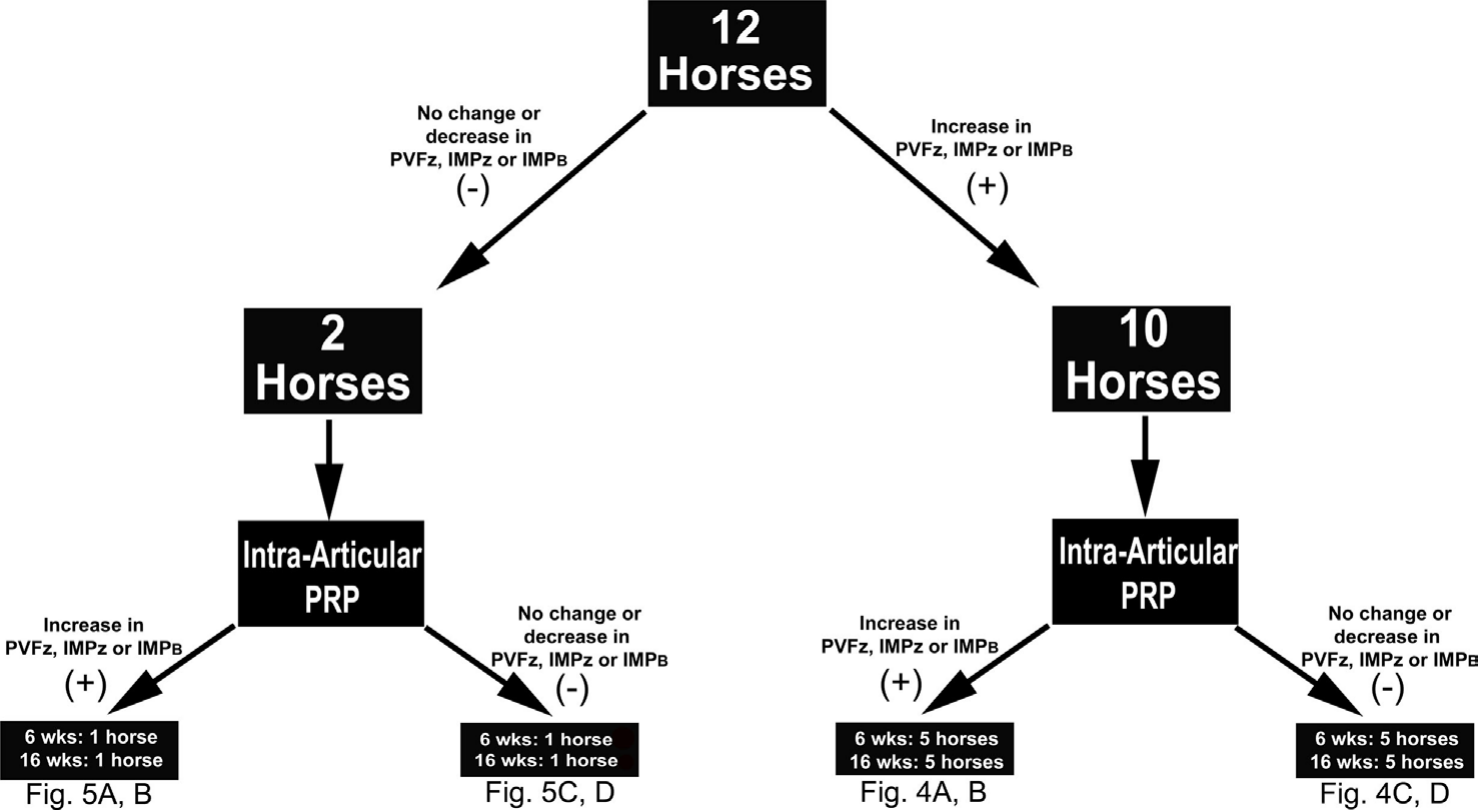
**Study design schematic indicating the number of horses that did (+) or did not respond (−) to intra-articular anesthesia (IAA) according to changes in the indicated kinetic variables**. Based on the same criteria, the number of horses that did (+) or did not (−) respond to intra-articular platelet-rich plasma (PRP), 6 or 16 weeks after administration, within each IAA cohort are also shown. Abbreviations: PVF_z_, peak vertical force; IMP_z_, vertical impulse; IMP_B_, breaking impulse.

A comprehensive kinetic gait analysis was performed on all horses (below) to determine the effect of IAA and PRP on 10 gait variables of each of the four limbs (Table 1). The changes observed after IAA served as the study standard for comparing the changes following PRP. It was anticipated that subjects that had detectable changes in gait variables after IAA would have similar, detectable changes in gait variables after PRP therapy (responders). Those subjects that did not have changes in gait variables after IAA were not expected to have detectable alterations after PRP therapy (non-responders) (Figure 1).

### Kinetic Gait Analysis

Kinetic gait data were collected as previously described (12) immediately prior to IAA, 20–25 min after IAA, 4 weeks after IAA (immediately prior to PRP administration), and 6 and 16 weeks after PRP therapy. A 900 mm × 900 mm force platform (Model #BP900900, Advanced Mechanical Technology, Inc., Watertown, MA, USA) embedded in the center of a 40-m concrete runway was used for all trials. The force platform surface is the same color and texture as the runway. An experienced handler trotted all horses for all trials. A trial was considered successful if a forelimb contacted the force platform followed by contact of the ipsilateral hind limb at a velocity of 2.00–3.80 m/s. A force of 50 N triggered data acquisition. Trials were not included if the hoof was not straight and completely on the platform or was within 5 cm of the platform edge (13). A series of five retroflective photocell sensors (Mek92-PAD, Joslyn Clark Controls, Inc., Lancaster, SC, USA) were used to determine velocity and acceleration in each trial. There is little difference in velocity between lame and sound horses (14), so horses were allowed to trot at a comfortable pace with no specific speed imposed on them for trials (12). However, because of the effect of velocity on ground reaction forces (15), the trials used for analysis were selected based on vertical force step cycle graphs (newton per kilogram versus time) that varied <5%. All trials were recorded at a rate of 1000 Hz and processed with commercially available software (Acquire v7.3, Sharon Software Inc., Dewitt, MI, USA). Four to five trials were included for the left and right limbs of each horse.

### Intra-articular Anesthesia

Joints were clipped and aseptically prepared with alternating 70% isopropyl alcohol and 2% chlorhexidine scrubs. Between 5 and 10 mL of 2% mepivacaine hydrochloride (Pfizer, Inc., New York, NY, USA) was administered to each joint. Volumes varied based on joint distention. If positive pressure was experienced, fluid administration was stopped. In cases of severe joint effusion, 3–5 mL of joint fluid was removed prior to injection. After the gait trial, compressive bandages consisting of a sterile, non-adhesive pad, rolled cotton, and adhesive bandage tape were applied over the treated joints. Horses were confined to stalls for 2 days prior to bandage removal and return to pasture housing. Complete physical exams were performed every 12 h while horses were confined to stalls.

### Platelet-Rich Plasma Treatment

Four weeks after IAA, autologous PRP was prepared with a commercially available kit (E-PET, Pall Corporation, Port Washington, NY, USA), according to the manufacturer’s instructions using solutions provided. Briefly, the filter system was preloaded with 9 mL of hypotonic capture solution (sterile water). Next, 55 mL of blood was aseptically collected from the jugular vein into a 60-mL syringe containing 5 mL of anticoagulant citrate dextrose solution. The blood–anticoagulant mixture was then added to the filter system that was, subsequently, gently inverted at least 10 times to mix the solutions. Using gravity flow, the mixture was filtered through the system over about 10 min. Next, 8 mL of a hypertonic harvest solution (Purecell™) was flushed through the filter into a separate sterile syringe over 2–4 s. The final isolate consisted of the filtrate in the proprietary harvest solution. Kit solutions were not individually injected into any treated joints.

Complete blood cell counts were performed (ANTECH Diagnostics, Irvine, CA, USA) on blood and filtrate aliquots (1 mL) collected in ethylenediaminetetraacetic acid coated vacutainer tubes. Immediately after preparation, 5–10 mL of PRP was injected into joints identically to IAA administration. Bandages were applied to joints and horses housed in stalls for 2 days prior to return to pasture. One veterinarian performed all procedures and monitored joints and vital parameters every 12 h during the stall confinement.

### Data Reduction

Gait variables included PVF_Z_, IMP_Z_, PFB, PFP, IMP_B_, IMP_P_, ML, MU, L_max_, and U_max_ (Table 1). All values were normalized to subject weight (newton per kilogram). Percent change was calculated as [(treatment value − baseline value)/baseline value] × 100. Baseline values were those collected prior to IAA or PRP, and treatment values were those immediately after IAA or 6 or 16 weeks after PRP. Responders were defined as those with an increase of ≥5% over baseline in PVF_Z_, IMP_Z_, or IMP_B_ of the affected limb after either treatment (see Results below for additional explanation).

### Statistical Analysis

Mean percent change after IAA was determined for all gait variables of each limb in all horses. Those limb variables with the highest prevalence of improvement among subjects that responded to IAA were identified. Changes in platelet concentration after filtration were evaluated with Student’s paired *t*-tests. Unpaired *t*-tests were used to compare OA scores between horses that did or did not respond to IAA and PRP. Associations between PRP response and IAA response, platelet concentration, and OA severity were determined with odds ratio tests. Relationships between kinetic variable changes after IAA versus PRP at each time point were evaluated with Pearson or Spearman’s rank order correlation tests based on the D’Agostino–Pearson omnibus normality test. All analyses were performed using commercially available software (SAS v9.4, Statistical Analysis Services, Cary, NC, USA; GraphPad Prism v6, GraphPad Software, Inc., La Jolla, CA, USA), and significance was considered at *P* < 0.05.

## Results

### Study Subjects

Seven geldings and five mares were included in the study [521.7 ± 50.1 kg; 7.5 ± 3.3 years (mean ± SD)] (Table 3). Breeds included 11 Thoroughbreds and 1 American Paint. There was no evidence of systemic or local inflammation associated with intra-articular injections based on physical examinations during stall confinement and daily assessments during pasture housing.

**Table 3:** **Radiographic osteoarthritis (OA) score, platelet-rich plasma (PRP) platelet and white blood cell (WBC) concentrations, and response to intra-articular anesthesia (IAA) and PRP of study horses**.

Breed	Age (years)	Weight (kg)	OA score	Platelet concentration (Plt/μL)	WBC concentration (WBC/μL)	Response to IAA	Response to PRP	
							6 weeks	16 weeks
Tb	6	432	4	8.97 × 10^5^	1.16 × 10^4^	Yes	Yes	Yes
Tb	4	496	4	7.23 × 10^5^	1.28 × 10^4^	Yes	Yes	Yes
Tb	3	472	3	5.20 × 10^4^	3.20 × 10^3^	Yes	No	No
Paint	15	536	3	2.50 × 10^5^	2.90 × 10^3^	Yes	No	No
Tb	6	579	4	7.98 × 10^5^	1.31 × 10^4^	Yes	No	Yes
Tb	9	476	4	6.63 × 10^5^	1.51 × 10^4^	Yes	Yes	No
Tb	10	572	4	6.21 × 10^5^	1.21 × 10^4^	Yes	No	No
Tb	4	550	3	9.54 × 10^5^	9.07 × 10^3^	Yes	No	Yes
Tb	7	480	4	7.20 × 10^5^	1.36 × 10^4^	No	No	No
Tb	7	551	3	2.69 × 10^5^	6.98 × 10^3^	Yes	Yes	Yes
Tb	9	523	4	7.02 × 10^5^	1.35 × 10^4^	No	Yes	Yes
Tb	10	592	5	7.85 × 10^5^	1.40 × 10^4^	Yes	Yes	No

### Radiographic OA

**Figure 2:**
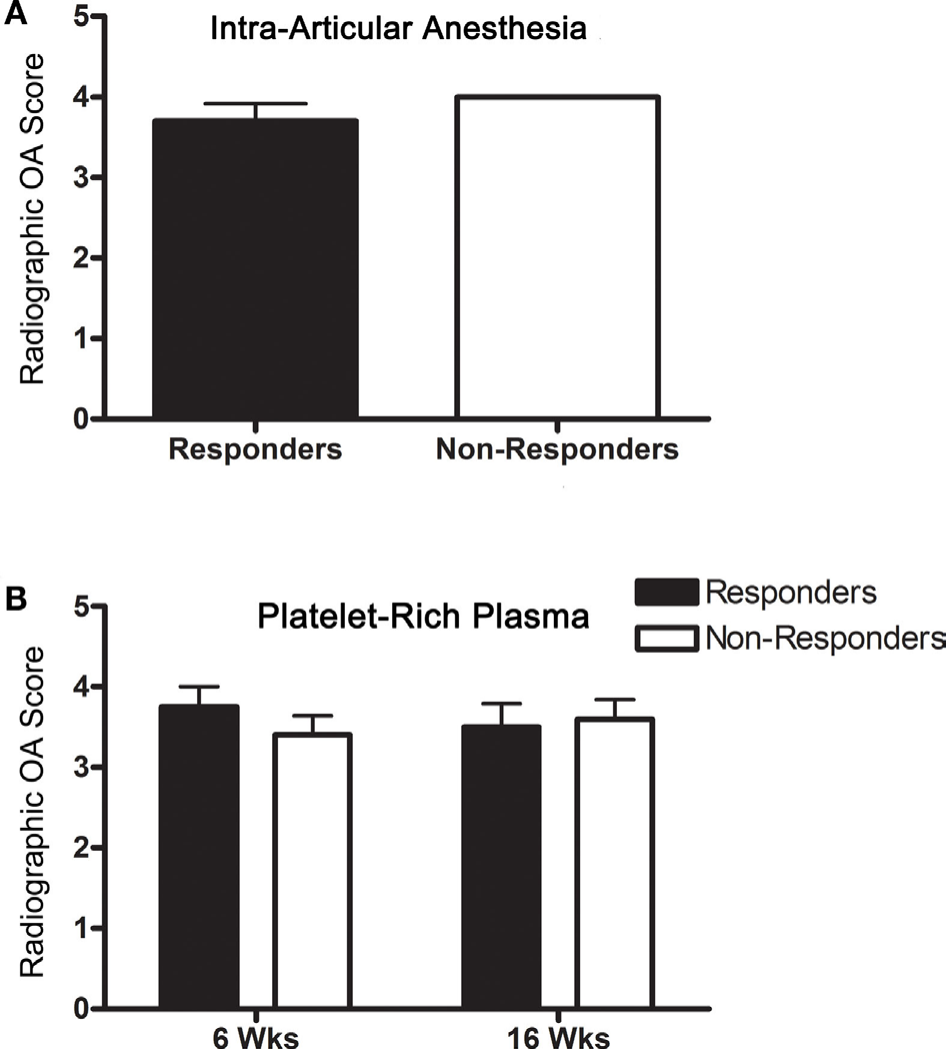
**Radiographic OA scores (mean ± SD) for responders and non-responders (A) to IAA and (B) PRP (6 and 16 weeks)**.

All horses had moderate to severe radiographic OA, and score did not change in any horse over the course of the study (Table 2). OA scores were not significantly different between IAA (Figure 2A) and PRP (Figure 2B) responders versus non-responders. The OA severity and response to PEP were not significantly associated.

### Kinetic Gait Analysis

Ground reaction force variables for each of the four limbs did not change in a detectable pattern among horses that showed reduced lameness after IAA (8, 9); however, increases in PVF_Z_, IMP_Z_, and IMP_B_ of ≥5% of the affected limb were most prevalent. For purposes of this study, a positive response to IAA or PRP was defined as ≥5% increase in one or more of the variables (PVF_Z_, IMP_Z_, or IMP_B_) of the affected limb. The relationships between gait variable changes of the affected limb after IAA and PRP were not significant 6 or 16 weeks after PRP treatment.

### Intra-articular Anesthesia

**Figure 3:**
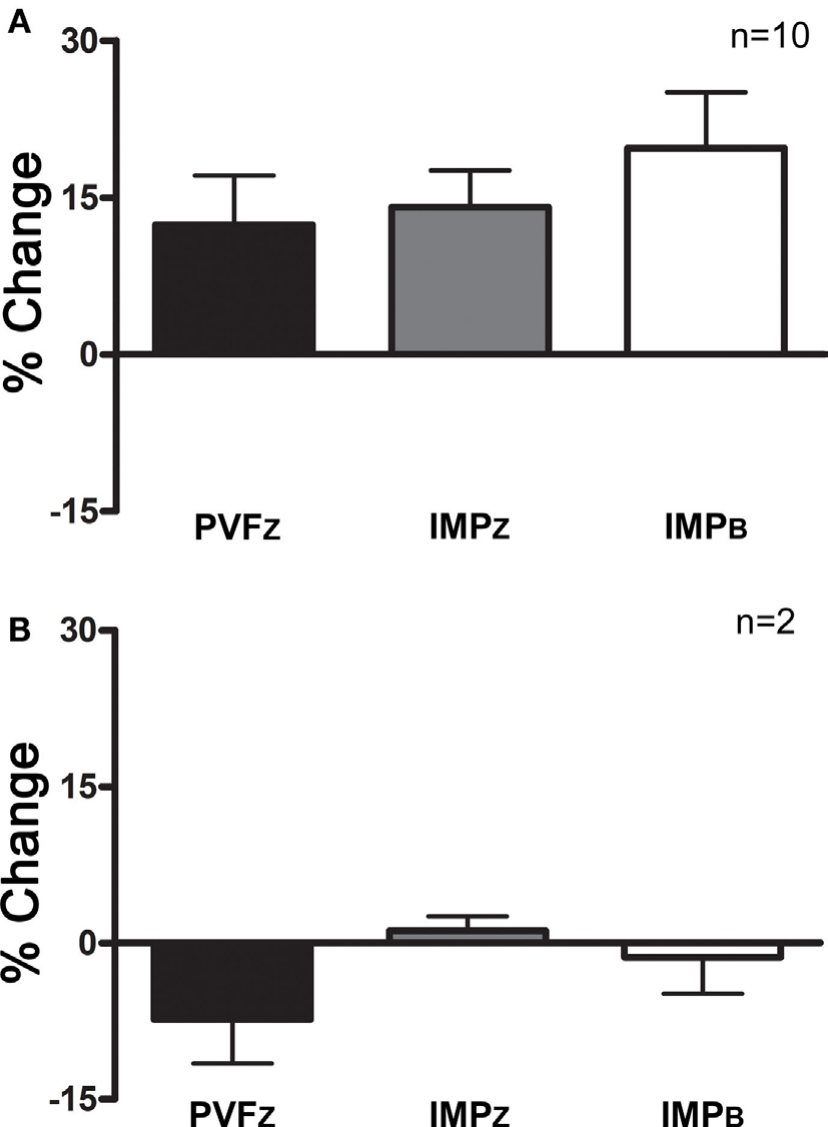
**Percent change (mean ± SD) in PVF_Z_, IMP_Z_, and IMP_B_ of the affected limb for (A) horses that responded to IAA and (B) horses that did not respond to IAA**.

Using the definition of a positive response above, 10/12 horses responded to IAA (Figure 3).

### Platelet-Rich Plasma Treatment

**Figure 4:**
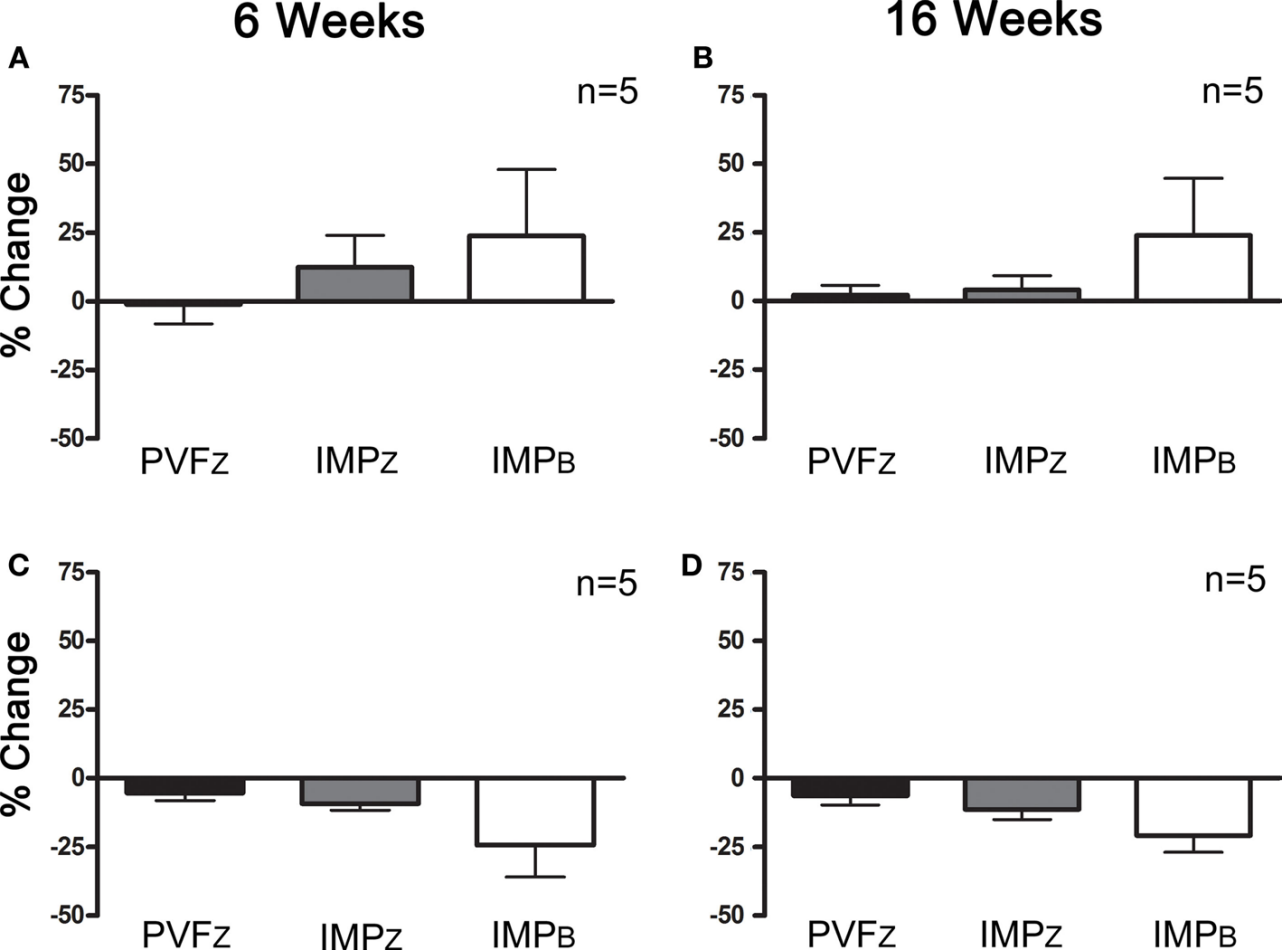
**Percent change (mean ± SD) in PVF_Z_, IMP_Z_, and IMP_B_ of the affected limb for horses that responded to IAA and (A) PRP after 6 weeks, (B) PRP after 16 weeks, (C) did not respond to PRP after 6 weeks, and (D) did not respond to PRP after 16 weeks**.

**Figure 5:**
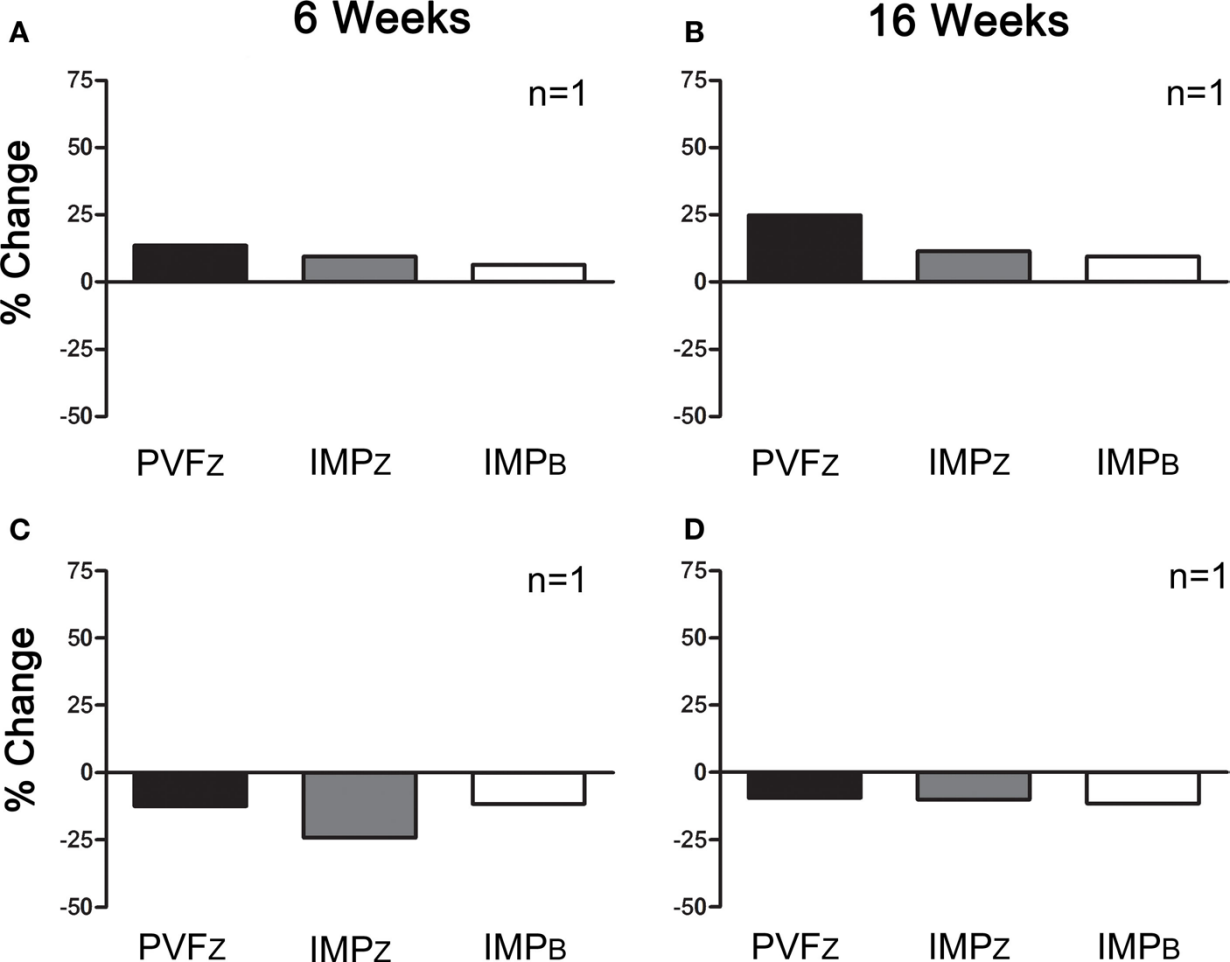
**Percent change (mean ± SD) in PVF_Z_, IMP_Z_, and IMP_B_ of the affected limb for horses that did not respond to IAA and (A) responded to PRP after 6 weeks, (B) responded to PRP after 16 weeks, (C) did not respond to PRP after 6 weeks, and (D) did not respond to PRP after 16 weeks**.

Within those horses that responded positively to IAA (*n* = 10), three responded positively to PRP 6 and 16 weeks after administration, and four responded at one time point only, either 6 or 16 weeks (Figure 4). Of the horses that did not respond to IAA, one responded to PRP treatment at both time points (Figure 5). The association between response to IAA and response to PRP was not significant.

### Effect of Platelet Concentration

**Figure 6:**
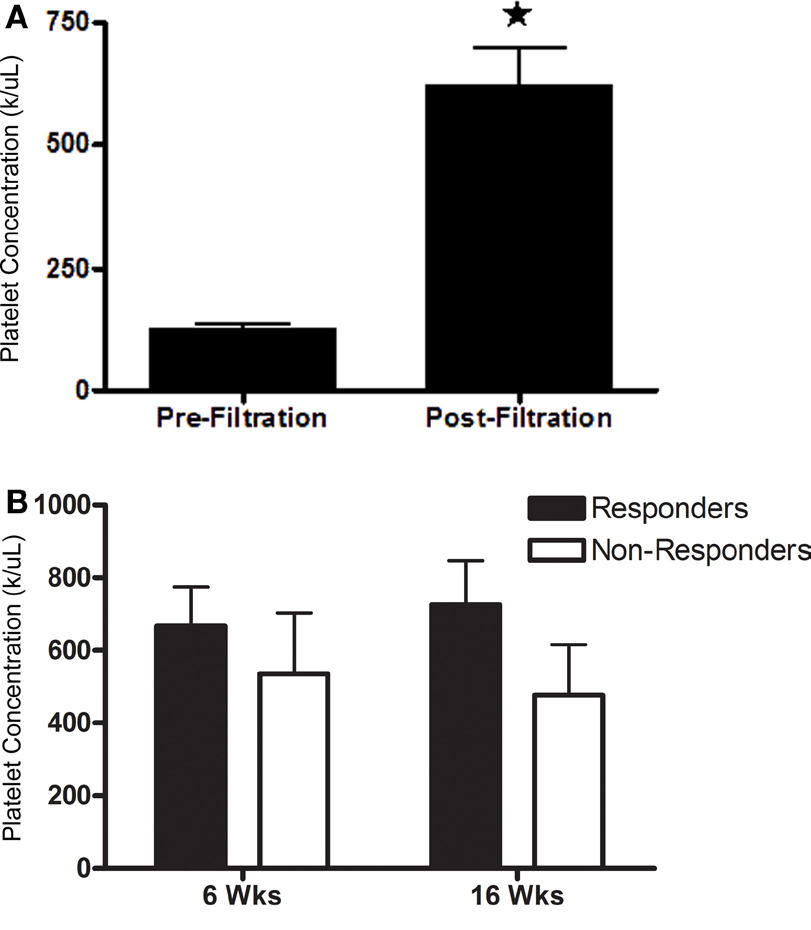
**Platelet concentration (mean ± SD) (A) prior to and after filtration and (B) administered to horses that responded (responders) or did not respond (non-responders) to intra-articular PRP 6 or 16 weeks after administration**. Columns with different symbols within graphs are significantly different from each other (*P* < 0.05).

Post-filtration platelet concentration ranged from 5.2 × 10^4^ to 9.5 × 10^5^ platelets/μL, a 1.3- to 9.1-fold increase. Mean platelet concentration was significantly higher after filtration (Figure 6A; *P* < 0.0001). Platelet concentrations were not significantly different between horses that had a positive response to PRP versus those that did not respond at either time point (Figure 6B). There was no significant association between platelet concentration and response to PRP.

## Discussion

The comprehensive gait analysis performed before and after IAA in this investigation established a baseline to assess response to PRP in a population of horses with naturally occurring, forelimb OA. Given the inherent variability among horses with a naturally occurring condition, we sought to identify a reproducible mechanism and a standard to compare response to intra-articular therapy based on current understanding of gait modifications associated with progressive, degenerative joint disease. Subjective lameness evaluations are limited by inter- and intra-observer error (10, 16), and there is evidence of overestimation of changes in lameness after treatment (17). Kinetic gait analysis is an established mechanism to overcome many of the limitations (14). Analgesia from IAA used for comparison in this study was not necessarily an achievable standard for intra-articular therapy since the pain relief 6 and 16 weeks after administration is unlikely to reach that of complete tissue desensitization. As an additional comparison, true negative controls, or horses that did not respond to joint desensitization and would therefore be unlikely to respond to joint pain relief associated with PRP, were included in the study. Based on objective gait analysis of horses with forelimb OA that did and did not respond to IAA, response to intra-articular PRP varied among horses with naturally occurring, forelimb OA.

Quantification of kinetic gait changes in multiple limbs makes it possible to detect compensatory gait changes (9). Evidence supports that it is necessary to quantify numerous gait variables to assess complex kinetic gait alterations (2). These considerations were the rationale to evaluate multiple ground reaction force variables in all limbs. However, the horses in this study did not respond to IAA with a consistent pattern of gait changes as expected from responses of horses with pain in only one limb. This is not surprising given the age of the population and the presence of significant OA in a forelimb joint. While horses were subjectively lame in one forelimb, it is likely that they experienced other sources of discomfort. As such, the limb and gait variables that consistently reflected improved gait in the population were selected to compare gait alterations following PRP. Although peak breaking force and braking impulse are less commonly used to quantify equine lameness, they correlate well to lameness severity (2) and superficial digital flexor tendonitis pain (18).

The equine population used in this study had joint changes that were likely beyond those typically considered consistent with continued use. Several available studies support that PRP treatment appears to be more effective in younger patients, human and equine, with mild cases of OA (19, 20). The population in this study was selected with the goal to ensure consistent forelimb lameness across trials to reduce potential variability or alternating lameness that could obscure the ability to discern treatment response. There are many horses with comparable OA that are “pasture sound” or appropriate for light work. These horses could benefit from a long-lasting, intra-articular therapy to reduce pain associated with OA. Hence, while the population was not consistent with high-performance horses, they represent a significant patient population.

Joint changes associated with iatrogenic injury are often used to evaluate therapeutic efficacy of autologous products (21). Such models are more closely representative of acute, traumatic OA in an otherwise healthy joint environment (7). The majority of clinical cases are progressive OA (1), a scenario that is best replicated through naturally occurring disease (22). Additionally, gait is often assessed with one kinetic gait variable in a single limb or via subjective evaluation (19, 23). These distinctions, among others, could account for the differences in results among studies. Additionally, a challenge of naturally occurring OA is that cartilage damage and joint environments can vary widely among joints with comparable radiographic changes (24, 25). Synovial fluid analysis may have provided additional information about joint inflammation; however, direct observation is required to assess differences in degenerative joint changes (26). Hence, variable responses among horses with seemingly similar radiographic OA may be attributable to significant differences in the local joint environment.

The duration of autologous platelet treatments is variable. Some reports indicate that treatment effectively reduces lameness in horses up to 8 months (23). Others suggest a decline in efficacy between 12 and 52 weeks after therapy, with most significant improvement between 7 and 14 days (19). Likely, the duration of response is directly related to the joint condition, including articular and periarticular tissue changes. As such, it is possible that the peak effect of the treatment was not captured at the 6- and 16-week time points in this study. The initial assessment point was selected to replicate reasonable therapeutic expectations in that more frequent injections, within 6 weeks, may not be physically reasonable or financial feasible. The highly inflamed joint environment, consistent with advanced OA, may shorten the effect of PRP, so earlier assessment points may have made it possible to identify a response that was not detected in this study. The 16-week time point was included to evaluate the duration of detectable effects in individual animals to help guide treatment strategies. More frequent assessments over the course of the study might have made it possible to determine a window during which treatment was effective for a greater number of horses.

A potential advantage of the system evaluated in this study is the fact that PRP can be isolated without the need for centrifugation, making “stall side” PRP preparation possible. Overall platelet concentrations were significantly higher after filtration, but numbers varied among preparations. There is no therapeutic standard for platelet concentration in PRP preparations (5). A proposed target concentration is around 1 × 10^6^ platelets/μL or a threefold to fivefold increase over whole blood, though studies have shown successful treatments with both higher and lower concentrations (27, 28). The potential effect of the white blood cell (WBC) concentration on PRP benefits is also a contemporary question with no clear answer. At present, a maximum concentration of 3 × 10^3^ WBC/μL is thought to avoid inflammatory cytokine accumulation (5). The majority of platelet concentrations in this study was consistent with current standards and reports using the same filtration kit (29), and there was no detectable effect of platelet concentration on treatment response.

Use of platelet products in combination with cell isolates has been tested both *in vitro* (30) and *in vivo* (31). Platelet lysate reportedly increases proliferation of equine adult multipotent stromal cells (MSCs) from adipose tissue *in vitro* (30), potentially due to high concentrations of growth factors (32). However, at high concentrations, platelet lysate decreases equine cord blood MSC proliferation *in vitro* (33). An investigation to evaluate the impact of autologous platelet-enriched fibrin scaffold with or without bone marrow-derived multipotent stromal cells (BMSCs) on equine stifle cartilage repair did not show a benefit of BMSCs (31). Another showed improved healing and lower inflammatory infiltrate in experimental equine tendon lesions treated with adipose-derived MSCs in platelet concentrate versus phosphate-buffered saline alone (34). The information highlights ongoing efforts to test and institute novel treatment options using progenitor cells and platelets to augment the efficacy of either alone.

This comprehensive evaluation of autologous PRP therapy in a population of horses with naturally occurring, forelimb OA provides unique information owing to the study design. Horses selected for inclusion based on specific criteria were treated, assessed, and housed under identical conditions throughout the study period. Additionally, gait changes were evaluated with quantifiable, kinetic measures. Potential kinetic changes were compared not only to a pretreatment baseline but also to a “gold standard” of joint anesthesia for each horse. Finally, horses that did and did not show kinetic gait changes associated with joint anesthesia were treated with PRP to test the potential for improvement unrelated to pain reduction in the treated joint. Based on the study findings, the first hypothesis is rejected. Kinetic gait variables did not change consistently in horses with forelimb OA following IAA. Nonetheless, meaningful kinetic variables for individual assessment of gait changes 6 and 16 weeks after PRP administration were identified for the study population. Similarly, the second hypothesis is rejected since kinetic gait variables changed in the same magnitude and direction in less than half of the horses after IAA and PRP administration. The outcomes suggest that there may be a potential benefit of autologous, intra-articular PRP therapy for forelimb OA in some horses. Additional work, including double-blind, randomized studies with large numbers of participants and more frequent observations over longer durations, is required to determine specific characteristics of degenerative joint disease that may influence the clinical effects.

## Conclusion

Together, the results of this study indicate variable changes in kinetic gait parameters following intra-articular administration of autologous PRP in horses with naturally occurring, forelimb OA.

## Ethical Considerations

This study was approved by the Louisiana State University Institutional Animal Care and Use Committee (protocol #10-092).

## Author Contributions

ML – contributed to the study design, data collection, data reduction, data analysis, data interpretation, preparation of the manuscript, and gave final approval of the manuscript. MM and PB – contributed to the study design, data collection, preparation of the manuscript, and gave final approval of the manuscript. HR and MK – contributed to data reduction, data analysis, preparation of the manuscript, and gave final approval of the manuscript. NR – contributed to data collection, data reduction, data interpretation, preparation of the manuscript, and gave final approval of the manuscript.

## Conflict of Interest Statement

Research funding and platelet filtration kits were provided by Pall Corporation. The authors declare that the funding partners approved the design prior to study initiation but did not participate in data collection, reduction, analysis, or interpretation. The manuscript contents, including the conclusion, are solely those of the authors.

## Supplementary Material

Figure S1 **Radiographs of the treated joint (two views) of each study subject**.
