# Supplementary figures and images for: Gait Changes Vary among Horses with Naturally Occurring Osteoarthritis Following Intra-articular Administration of Autologous Platelet-Rich Plasma
Source: Front Vet Sci. 2016 Apr 13;3:29. doi: 10.3389/fvets.2016.00029 (PMC4829588; doi:10.3389/fvets.2016.00029)

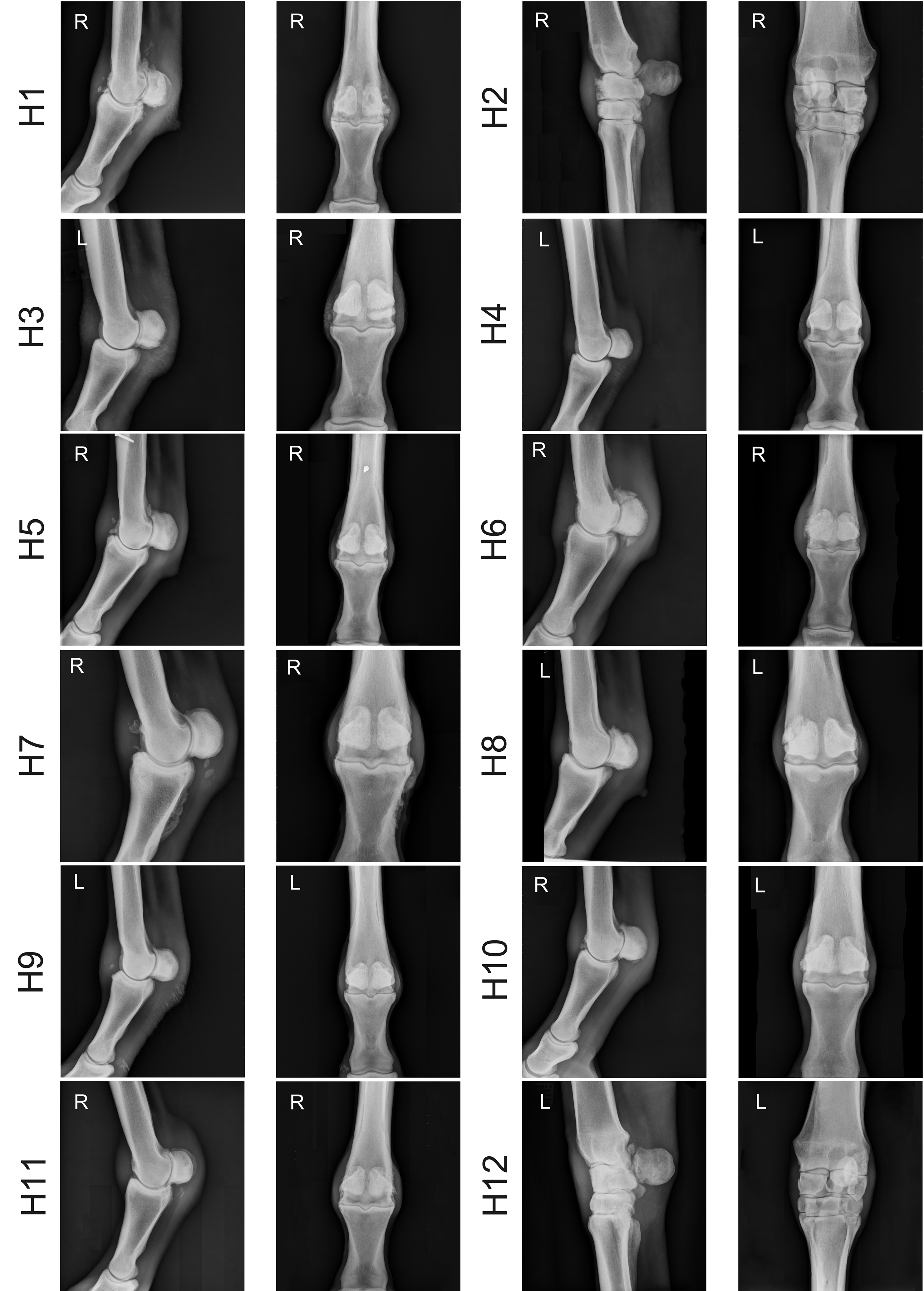

Supplement: Figure S1 — Radiographs of the treated joint (two views) of each study subject. [file Image_1.TIF]
